# Understanding Physiology and Impacts of High Temperature Stress on the Progamic Phase of Coconut (*Cocos nucifera* L.)

**DOI:** 10.3390/plants9121651

**Published:** 2020-11-26

**Authors:** K. B. Hebbar, P. Neethu, P. Abhin Sukumar, M. Sujithra, Arya Santhosh, S. V. Ramesh, V. Niral, G. S. Hareesh, Paingamadathil Ommer Nameer, P. V. V. Prasad

**Affiliations:** 1 ICAR-Central Plantation Crops Research Institute, Kasaragod 671 124, Kerala, India; neethupsankaran@gmail.com (P.N.); abhinsukumarp@gmail.com (P.A.S.); sujithra.m@icar.gov.in (M.S.); aryaaromal36@gmail.com (A.S.); ramesh.sv@icar.gov.in (S.V.R.); niral.v@icar.gov.in (V.N.); hareesh.gs@icar.gov.in (G.S.H.); 2 Academy of Climate Change Education and Research (ACCER), Kerala Agricultural University, Vellanikkara, Thrissur 680 656, Kerala, India; nameer.po@kau.in; 3 Department of Agronomy, Throckmorton Plant Science Center, Kansas State University, Manhattan, KS 66506, USA; vara@ksu.edu

**Keywords:** coconut, climate change, high temperature, pollen tube growth, progamic phase, stigma receptivity

## Abstract

The reproductive phase of coconut is extremely sensitive to high temperature, manifesting as button (female flower) shedding and poor nut set. The progamic phase, which elapses from pollination to fertilization, is one of the most critical phases during the sexual reproduction processes in annuals and fruit trees and is extremely vulnerable to high temperature. Hence, we investigated the progamic phase of the tall coconut cultivar West Coast Tall (WCT) and the effect of high temperature on the phase under both in vivo and in vitro conditions. Coconut has a long pistil and its length was found to be 18.2 ± 4.9 mm in WCT. Pollen germination on stigma occurred one day after pollination and the pollen tube traversed through the pistil and reached micropyle of ovule four days after pollination at 29 °C. However, high temperature (T_max_ > 33 °C), both under in vivo and in vitro conditions, significantly reduced pollen tube growth through the pistil, suggesting its inability to reach the ovule on time to effect fertilization. High temperature also advanced nectar secretion and stigma receptivity and the receptive stigma was dry without nectar, rendering it unappealing to insect pollinators. Thus, both poor pollination and the inability of pollen tube to reach the ovule on time to effect fertilization could be the cause of poor nut set in the coconut variety WCT under high temperature. However, it was encouraging to note that the pollen tube growth was less vulnerable to elevated temperature under high humidity, suggesting that climate change effect on coconut in coastal regions with high humidity might be less severe.

## 1. Introduction

Coconut (*Cocos nucifera* L.) is one of the important tropical crops cultivated extensively in 12.08 million ha in 92 countries with an annual production of 69 billion nuts [1]. Coconut provides food security and livelihood opportunity to 20 million people globally and 10 million people in India through cultivation, processing, marketing and trade-related activities. Thus, coconut value chain has profound influence on rural economies around the world. Coconut can be successfully grown in areas where the annual rainfall is 1300 mm or above [2], under the prevalence of high humidity, at an optimum temperature between 27 °C and 30 °C and on moderate to well-aerated soils [3]. In India, coconut cultivation is mostly restricted to the west coasts of Karnataka and Kerala where the annual rainfall is >2000 mm and is grown at a T_max_ (maximum temperature) of 34–36 °C for few hours during day time, while in the east coast of Andhra Pradesh and Tamil Nadu where the rainfall is low (around 1000 to 1200 mm), T_max_ reaches as high as 40–42 °C for a few months during summer [4]. As per climate change projections, temperature is likely to increase further in these regions by 1.1–2.6 °C by the end of the 21st century [5,6]. As coconut flowers throughout the year and almost produces one inflorescence per month, its likelihood of getting exposed to the spells of high temperature is very high.

Temperature is a major determinant of nut yield in coconut [7]. Coconut is not only sensitive to high temperature stress at the seedling stage [8,9], but also at the reproductive stage where in shedding of buttons or young female flowers is a common phenomenon under high temperature and has great bearing on the yield of coconut [10,11].The temperature prevailing at the time of fruit (nut) setting profoundly influences the number of nuts set per inflorescence [12]. High temperature days (T_max_ ≥ 33 °C during day time) during the first three months of inflorescence opening severely reduce the nut set of an inflorescence [11]. In Sri Lanka, inflorescences opened during the months of January, February and March [11] and in India during the months of April and May [10] had very poor nut set because it coincides with a greater number of days with high temperature (>33 °C). Coconut inflorescence or ‘spadix’ is monoecious, bearing both male and female flowers, but it is protandrous. The male flowers open, liberate pollen and fall off for a period of 19–25 days, and 2–4 days after cessation of the male phase, the pistillate (female) flowers become receptive. In the majority of the cultivated varieties of coconut, the asynchrony between the male and female phase warrants movement of pollen from one flower of either the same plant or a different plant to effect pollination [10,13] and thus making the process of fertilization more vulnerable to high temperature. Thus, high temperature outside the range of its tolerance reduces both pollination and fertilization in coconut, but the botanical and physiological basis of this phenomenon is not apparent.

Poor nut set is common in many cultivated and wild plant species, when subjected to high temperatures during their flowering phase. Instances of reduced fruit set are reported in temperate fruit trees like apricot (*Prunus armeniaca* L.) with an increase in the daily temperature during the week preceding anthesis [14], in peach (*Prunus persica* L. Batsch) due to high temperature one month before anthesis [15] and in sweet cherry (*Prunus avium* L.) owing to increased temperature following anthesis [16]. Disruptive effects of high temperature on the fruit set of annual crops have also been extensively investigated [17,18,19,20,21,22]. In many of the grain crops, the most sensitive reproductive stage to high temperature stress is during gamete development (pollen or ovule development), progamic phase (pollen viability, pollen germination, pollen tube growth rate, pollen tube dynamics, stigma receptivity and ovule viability) and embryo development [21]. The majority of these studies have focused on the high temperature effect on male gametes [18,23,24,25,26,27] and a few others have analyzed sporophytic generation from the post-zygotic stage to the reproductive phase [28,29,30,31]. Very few studied the progamic phase, which elapses from pollination to fertilization, is one of the most critical phases during the sexual reproduction process in plants and is extremely vulnerable to the prevailing environmental conditions [30,32,33]. The effect of temperature on the progamic phase has been comprehensively examined in many herbaceous species [34,35,36,37,38,39,40,41,42], woody species and tree crops [43,44,45,46,47,48,49,50,51,52,53,54,55,56].

In coconut, among the different stages of progamic phase, pollen germination with high temperature stress was well studied under in vitro. The mean cardinal temperatures for pollen germination of coconut genotypes ranged from 23.5 °C to 29.5 °C, 9.7 °C to 16.5 °C and 40.1 °C to 43.9 °C for T_opt_ (Optimum temperature, temperature at which maximum pollen germination is observed), T_min_ (Minimum temperature, temperature below which pollen does not germinate) and T_max_ (Ceiling temperature, temperature above which pollen does not germinate) respectively [57], while T_max_ values for pollen germination of the most tolerant and less tolerant hybrids were 41.9 °C and 39.5 °C, respectively [58]. However, there is no report delineating the in vivo progamic phase of coconut and its response to high temperature stress. The primary objective of this research was to understand the progamic phase of the coconut variety West Coast Tall (WCT) and its response to high temperature stress on stigma receptivity, pollen germination, pollen tube growth and pollen tube dynamics under both in vivo and in vitro conditions. This study was also aimed at devising a suitable in vivo and in vitro temperature induction methodology for exposing the female flowers of coconut to high temperature stress conditions without affecting its microclimate. We hypothesize that the progamic phase of coconut is sensitive to high temperature stress, resulting in poor pollen germination and pollen tube growth through pistil, resulting in poor nut set.

## 2. Results

### 2.1. Temperature of Experimental Site

**Figure 1:**
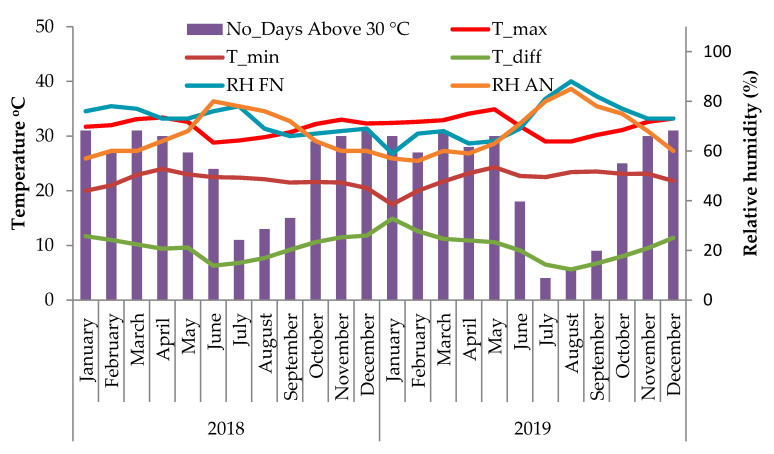
Fluctuations in monthly average maximum temperature (T_max_), minimum temperature (T_min_), difference between T_max_ and T_min_ (T_diff), days with T_max_ greater than 30 °C and relative humidity % in the forenoon (RHFN) and afternoon (RHAN) during the experimental period for the years 2018 and 2019.

The overall meteorological condition of the study site is presented in Figure 1. The in vivo studies were conducted in the April and May months of 2018 and 2019, during which the T_max_ of the experimental site was relatively high and a large number of days in these months had average T_max_ greater than 30 °C (Figure 1). The average T_max_ values during the months April and May were 33.4 °C and 32.63 °C in 2018 and 34.1 °C and 34.93 °C in 2019, respectively. The average T_min_ values for the same period were 24 °C and 23.3 °C in 2018 and 23.2 °C and 24.3 °C in 2019, respectively. In 2018, 30 and 27 days and in 2019, 28 and 30 days had average T_max_ greater than 30 °C during April and May months, respectively.

### 2.2. Progamic Phase of Coconut

**Figure 2:**
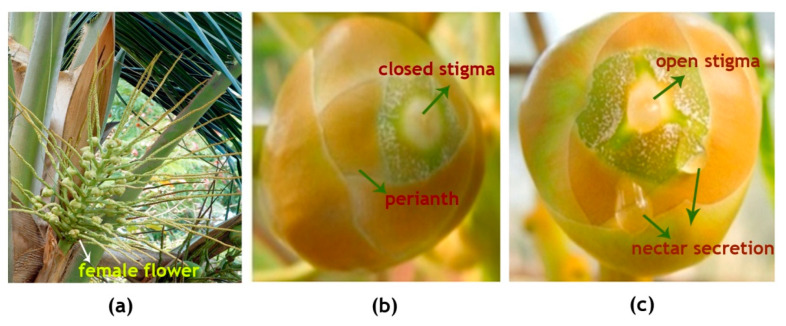
Fully opened inflorescence of the coconut variety West Coast Tall (WCT) showing (**a**) female flowers at the base of rachilla while all the male flowers were already shed,(**b**) a female flower few days before its receptivity and (**c**) a receptive female flower with moist open stigma and nectar secretion from the orifice.

The inflorescence of WCT contains on an average 20 ± 5 female flowers per spadix (Figure 2a: Table 1). The female flowers which are globose, pistillate flowers(buttons) are 2.5 ± 0.5 cm in diameter (Figure 2b) and the young calyx consisting of six thick, imbricating perianth lobes in two whorls is tightly folded over the pistil. The female flower, when receptive, attains more than double the size of what it was on the opening of the spadix, with the stigma expanded as three erect teeth (Figure 2c). The stigma is white in color and the nectar observed in the orifices starts to ooze 2–3 days before the opening of stigma tip (Table 1). Small drops of nectar were observed to be oozing out from the three orifices alternating with the three lobes of open stigma (Figure 2c).

**Figure 3:**
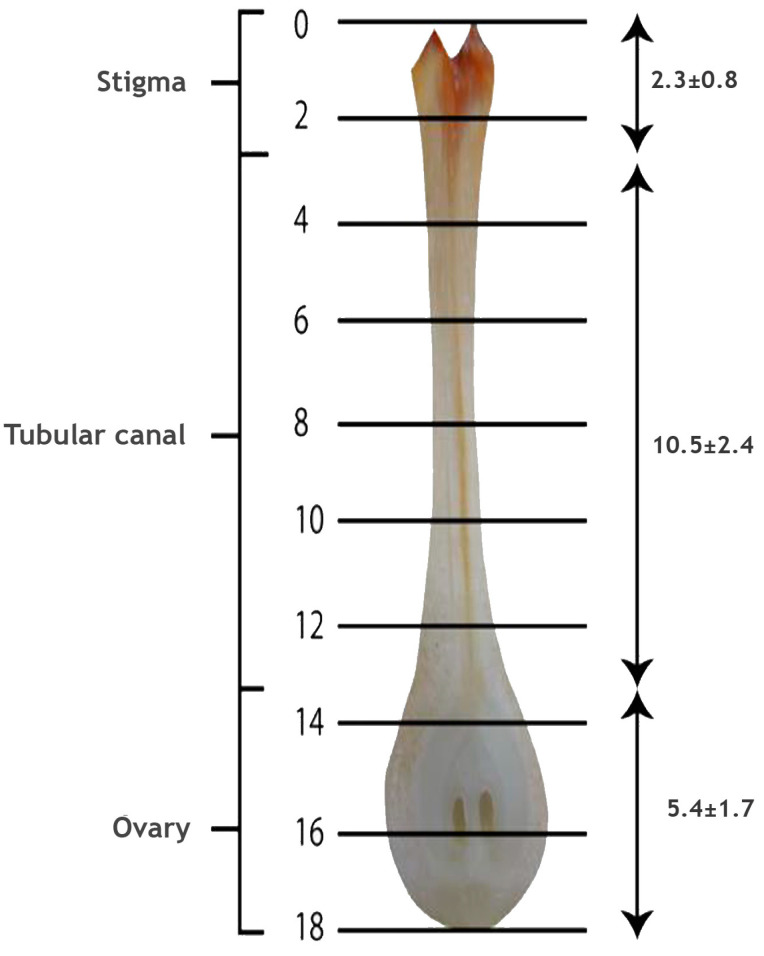
Longitudinal section of pistil of the coconut variety WCT. The cross lines from 0 to 18 indicate the length in mm (mean ± SD) of the stigma, tubular canal and ovule-bearing region.

The pollinated flowers (*n* = 6) of coconut on dissection were observed to have a large and fleshy pistil and ovary. The lengths of the stigma, tubular canal and ovule-bearing region of the female flower were 2.3 ± 0.8, 10.5 ± 2.4 and 5.4 ± 1.7 mm, respectively, and thus, the total length of the pistil in coconut was 18.2 ± 4.9 mm (Figure 3). The quadruple staining procedure developed by Alexander [59] adopted here had taken 24 h to fix the stain, and another two days for clearing and softening. Thus, the whole procedure from tissue preparation until the microscopic observations required four days at 45 °C. In the acid medium, both acid fuchsin and aniline blue were effective in staining the pollen grains, pollen tubes and canal (stylar) tissue in dark blue color. However, following the clearing and softening procedures, the dark stain absorbed by the canal tissue was removed and it appeared lavender or light pinkish color, and the differential staining of parts greatly assisted in tracing the pollen tube in pistil.

**Figure 4:**
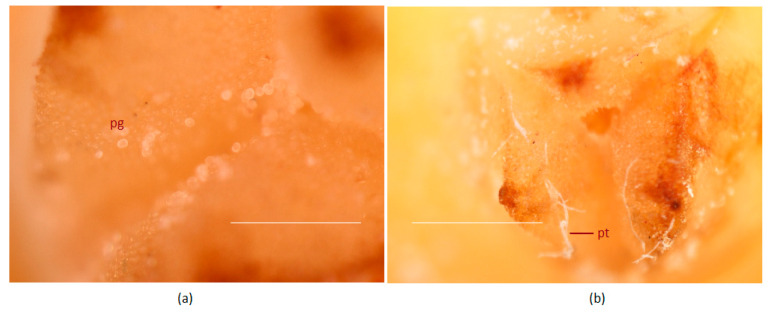
In planta pollen grain germination of the coconut variety WCT on the receptive stigma surface after pollination. (**a**) Ungerminated pollen grains 4 h after pollination and (**b**) germinated pollen grains with pollen tubes 24 h after pollination. Pollen tubes are marked by an arrow (pt); pg, pollen grains. Stigma surface was stained with quadruple stain [59] and visualized using a stereo microscope. Scale bar = 100 μm.

**Figure 5:**
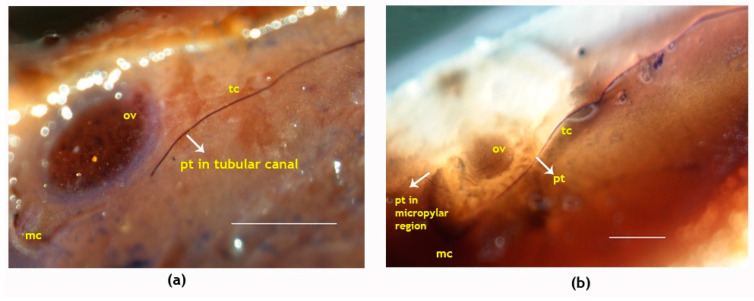
Longitudinal sections showing pollen tube growth in the pistil of the coconut variety WCT. (**a**) Pollen tubes growing inside the tubular canals three days after pollination and (**b**) pollen tubes accessing the micropylar region of ovule four days after pollination. Pollen tubes are marked by an arrow; pt, pollen tube; tc, tubular canal; ov, ovule; mc, micropyle. Sections of the pistil were stained with quadruple stains [59] and visualized using a stereo microscope. Scale bar = 100 μm.

With the dark blue stains, the pollen tubes were identifiable on the stigmatic region and could be traced along the entire length of the tubular canal connecting the stigma to the ovule-bearing region. Pollen germination occurred on receptive stigma and could be observed 24 h after pollination (Figure 4a,b) and during that period there was hardly any pollen tube expansion. On day two, pollen tube started expanding and traversed 3.2 mm through the canal (Figure 5, Table 2). Three days post-pollination, there was a sudden spurt in its growth and it reached 14 mm in length (Figure 5a) and on the fourth day, it could traverse the entire length of canal and was visible near the micropyle of the ovule (Table 2, Figure 5b). Thus, it took four days for the pollen tube to traverse from the stigma to ovule, a distance of 18.2 ± 4.9 mm, following the process of pollination.

### 2.3. Effect of High Temperature Stress on Progamic Phase In Vivo

**Figure 6:**
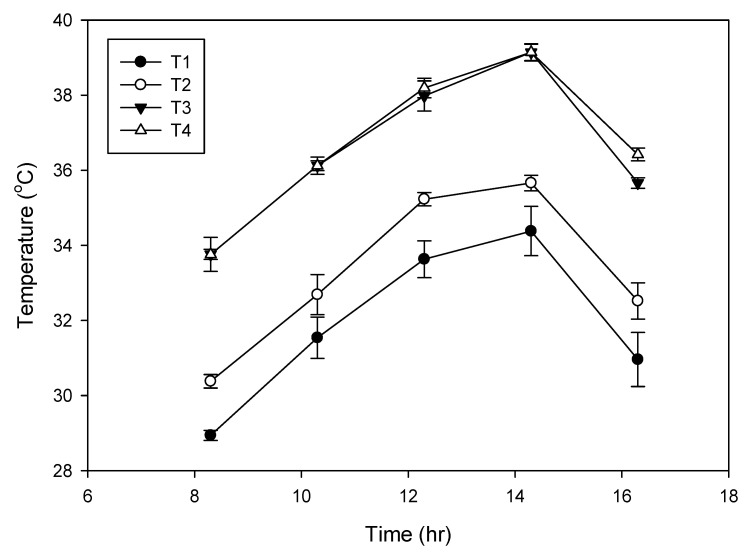
Mean diurnal temperature (*n* = 3) of the coconut inflorescence enclosed in transparent chamber with temperature treatments. T1, ambient; T2, chamber control;T3, chamber with 3 °C rise in temperature at an average relative humidity (RH) of 55 ± 5%; T4, chamber with 3 °C rise in temperature at a mean RH of 65 ± 5% during April 2018. Bars on the lines indicate mean ± SD (standard deviation).

Mean diurnal temperatures around the inflorescence for control and high temperature treatments are presented in Figure 6. In ambient condition, the T_max_ near the inflorescence was 29 °C (70% relative humidity (RH); Figure 1) at 09:00 and as the day progressed, it increased and peaked around 14:30 to 34 °C (60% RH; Figure 1) and later declined to 27 °C at 16:30. The enclosure of the heat chamber increased the temperature around the inflorescence by 1–1.5 °C and thus, the T_max_ was high and ranged from 30.3 °C to 35.6 °C (chamber control). In the elevated temperature treatment (3 °C above chamber control), inflorescence was consistently exposed to high T_max_ throughout the day which ranged from 33.7 to 39.14 °C. As the temperature during the treatments was raised, there was a drop in the average humidity level to 55 ± 5% as against the mean values of 65 ± 5% of ambient. Thus, the female flowers were exposed to temperature from 29 to 39 °C under in vivo conditions.

**Figure 7:**
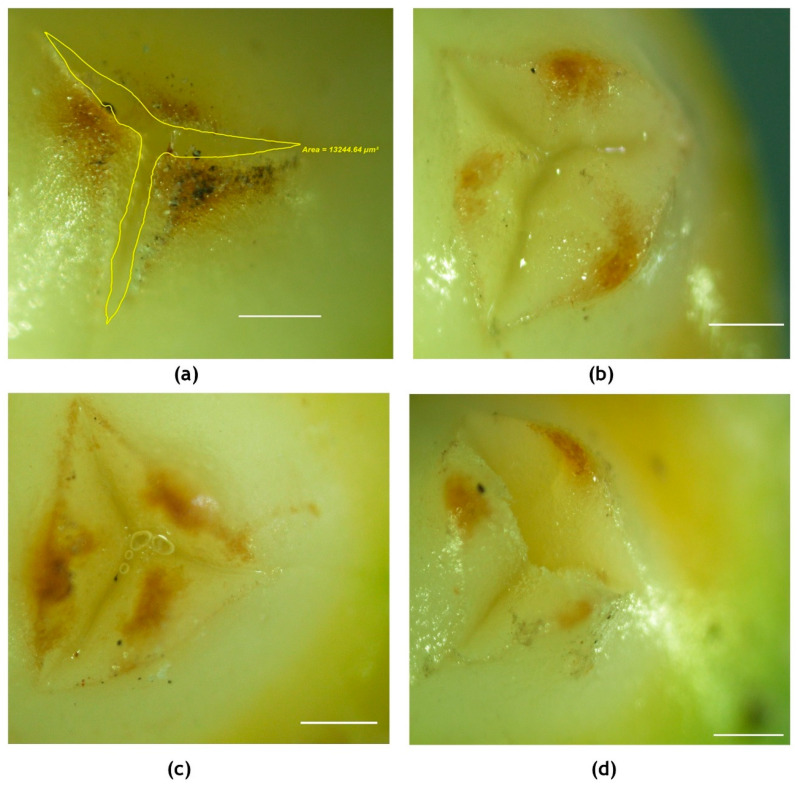
Visualization (2X) of receptive stigma of the coconut variety WCT under (**a**) ambient, (**b**) chamber control, (**c**) chamber with 3 °C rise in temperature and 55 + 5% RH and (**d**) chamber with 3 °C rise in temperature and 65 + 5% RH. The area in-between the opening of perianth lobes was measured as shown in (a) using a stereo microscope. Scale bar = 100 μm.

Visual observations revealed a marked effect of high temperature on the emergence of stigmatic region, nectar secretion and stigmatic appearance when the high temperature stress was imposed on the inflorescence six days before the receptivity of female flowers. Some of the changes observed are listed in Table 1. At 33.7 °C, nectar secretion was observed to start within a few hours of exposure to the high temperature, almost 5–6 days prior to receptivity, in contrast to 2–3 days in control plants (28 °C). The stigma of receptive flowers at 29 °C was white in color and moist, with a lot of nectar in the surrounding orifice (Figure 2c), while at 33.7 °C, it was dull and non-sticky and the surface was dry. In receptive stigma, the perianth lobes were wide open under ambient condition and had an area of 13,250.86 ± 109 µm^2^, while it was 8651.30 ± 127µm^2^ with chamber control, 2109.32 ± 78 µm^2^ with 3 °C rise in temperature (55 ± 5%RH) and 6527.08 ± 113 µm^2^ with 3 °C rise in temperature (65 ± 5% RH) (Figure 7). Additionally, as high as 28% of the flowers remained non-receptive at high temperature, whereas only 10% of the flowers were non-receptive in chamber control (Table 1). High temperature treatment effect was alleviated to a certain extent with the concomitant rise in humidity around the inflorescence to 65 ± 5%. The reasonably extended nectar production phase observed till the receptivity of stigmas prevented the premature drying of stigma to a large extent.

**Figure 8:**
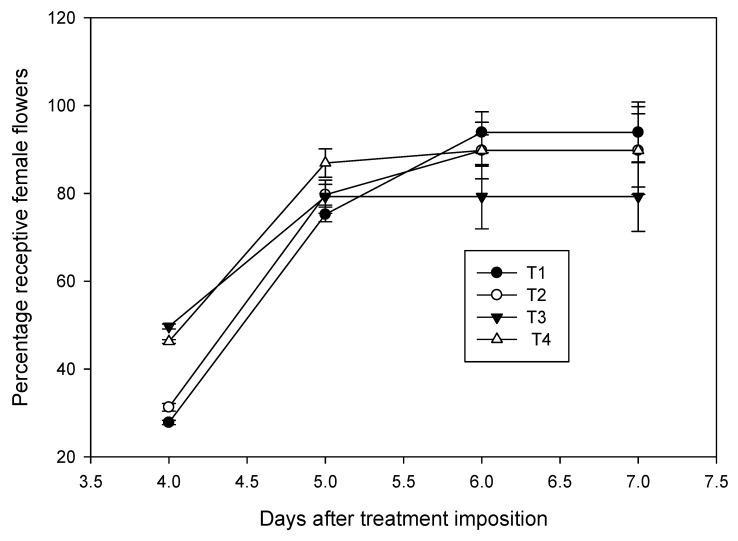
Effect of high temperature treatment on female flower receptivity of the field-grown coconut variety WCT. Receptivity of female flowers, which was denoted by splitting of white stigma and secretion of nectar, of ambient grown plants (T1) was compared with percentage decline in receptivity of flowers in chamber control (T2), chamber with 3 °C rise in temperature at 50 ± 5% RH (T3) and chamber with 3 °C rise in temperature at 65 ± 5% RH (T4). Bars on the lines indicate mean ± SD. Temperature treatment to the inflorescencewas imposed six days before the female flower receptivity.

Furthermore, a stimulatory effect of high temperature on female flower receptivity was observed during early stages (Figure 8). Inflorescences that opened at the same period, but exposed to high temperature for four days, had 46% receptive flowers compared with 31% receptivity in chamber control. Receptivity continued to increase, and it was 79% on 5th day for both the chamber control and high temperature treatments. Further increment in stigma receptivity was not observed with high temperature treatment; however, in control, it continued to increase to 89% on the 7th day. Interestingly, those inflorescences which were maintained at high humidity showed a minimal effect of high temperature and the receptivity was on par with that of control.

High temperature significantly reduced pollen tube expansion in pistil (Table 2). At 29 °C, pollen tube could traverse the length of 19.73-mm pistil in four days. During the same period, it reached only 12.89 mm at 30.3 °C and there was no expansion at 33.7 °C with RH 55 ± 5%. However, when the RH was raised to 65 ± 5%, a slow pollen tube expansion albeit of 1.88 mm was observed after four days.

### 2.4. Effect of High Temperature Stress on Progamic Phase In Vitro

The pollen tube required four days to traverse through the pistil, from stigma to ovule, to cover a distance of 19.9 mm under in vitro conditions (Table 3), which was in line with the in vivo observations. The mean pollen tube length along the canal region showed a significant reduction with the increasing temperature: at 31, 33, 35, 37 and 40 °C with 55% RH, the pollen tube could traverse only 8.64, 7.04, 1.22, 1.21 and 0 mm, respectively. On the other hand, when the RH of the chambers was raised to 65%, the pollen tube could traverse 19.15, 19.14, 9.88, 3.81 and 1.42 mm through the pistil for the same temperature treatments, respectively. Pollen tube growth was significantly reduced at absolute T_max_ > 33 °C under both the humidity levels.

## 3. Discussion

This research indicated that high temperature stress significantly influences the progamic phase (receptivity of stigma, pollen germination and pollen tube growth through pistil) of the coconut variety WCT and could be a cause for poor fertilization of coconut flowers. High temperature stress is one of the most critical factors affecting nut set in coconut [9,12]. Fruit setting is generally poor in those inflorescences whose opening coincides with the months of high temperature (>33 °C), January through March in Sri Lanka [11] and March through May in India [4]. In most of the field crops as well as in fruit trees, it was observed that the progamic phase of reproductive stage is the most sensitive for high temperature conditions [21,30,32], which is not understood in coconut. Moreover, the inherent problem of plant height (a few meters at flowering) is the major challenge of imposing the high temperature treatment to the whole tree of coconut. Therefore, our aim was to, in addition to understand the anatomy and kinetics of the progamic phase, devise a reliable in vivo and in vitro temperature induction methodology to expose the female flower to high temperature conditions without affecting the microclimate. 

Coconut is a monoecious crop; however, in WCT, the popular tall variety selected for this study, there is hardly any overlapping of the male and female phases within an inflorescence and hence, preponderance of cross pollination is observed [13]. WCT plants produce on an average one inflorescence per month and each inflorescence possesses 20 ± 5 female flowers. After 3 ± 1 days of cessation of the male phase (lasts for 19 ± 5 days post anthesis), female flowers of WCT become receptive, noticed by the protrusion of pistil through the perianth segments, resulting in a wide-open stigma moist with nectar. Under natural conditions, pollination in coconut is carried out by pollinators like honeybees as well as wind [13,60]. In this experiment, controlled pollination was effected by dusting WCT pollen on receptive stigmas to ensure the known source of pollen and to document the exact time and date of pollination. 

**Figure 9:**
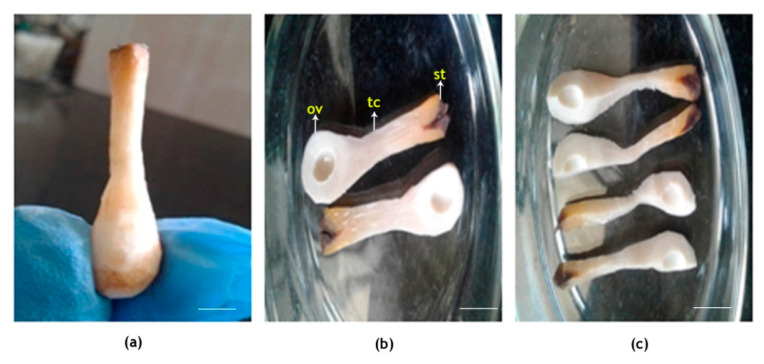
Dissection of coconut female flower. Outer calyx and exodermis completely peeled off, showing (**a**) intact longitudinal part; (**b**) two halves showing stigma, tubular canal and ovule-bearing region; and (**c**) small longitudinal sections. St, stigma; tc, tubular canal; ov, ovule. Scale bar = 5 mm.

The stigma, tubular canal and ovule-bearing region of the intact pistil of WCT (Figure 9) obtained after the dissection of pollinated flowers were of 2.3 ± 0.8, 10.5 ± 2.4 and 5.4 ± 1.7 mm in length, respectively, and thus, the total length of the reproductive part was 18.2 ± 4.9 mm. The diameter of the ovary is 8–10 mm [61]. Thus, the length of coconut pistil is quite large compared to most of the cultivated species but comparable to the pistils of *Passiflora* and Solanaceae species [62]. The longitudinal sections of the pistil were hard, but the quadruple staining used could differentiate the pollen tubes (dark blue) from pistil tissue (lavender or light pinkish) and hence, the pollen tubes were distinguishable from the stigmatic region and could be traced along the entire length of the pistil till it reached the ovule. Pollen germination on stigma, in vivo, occurred 24 h after pollination under ambient condition of 29 °C. Similarly, the pollen tube expansion on stigma was also slow initially (second day) and showed a sudden spurt in growth on the third day and reached the micropyle of the ovule, a distance of 19 mm in four days. In comparison, under the same temperature under in vitro growth medium, WCT pollen germination occurred within thirty minutes after placing it in the nutrient medium and maximum pollen tube expansion of 0.55 mm occurred within two hours [57]. The differential response could be attributed to the composition of the growth medium as the pollen tubes were growing under identical temperatures. In this context, the technique of inducing high temperature stress in vivo described in this study is critical to ascertain the actual effect of elevated temperature stress without altering other associated factors of progamic phase of coconut. Though pollen tube expansion takes relatively a long period in coconut than in most other species, it is comparable to species like pecan, pear and apple which require 3–4 days for the pollen tube to traverse through the pistil [63,64,65].

In order to decipher how high temperature affects the progamic phase in coconut, in vivo and in vitro experiments were conducted. The field site selected for the in vivo study had average monthly T_max_ of 29 °C in the morning (09:00) and around 32 °C in the afternoon (14:00) during the months of April and May. The ideal time for assisted pollination in coconut is from 08:00 to 11:00, coinciding with the receptivity of the female flowers [66] and thus, the ambient temperature of the site selected for the study was within the admissible range. As it was cumbersome to expose the whole flowering coconut plant to high temperature, enclosing the individual inflorescence with 20 ± 5 female flowers in a transparent chamber fitted with a microprocessor-controlled heater and humidifier could effectively simulate a high temperature effect on female flowers. With a rise in temperature inside the chamber (3 °C > chamber control and 4.7 °C > ambient), humidity dropped to 55% from ambient around 68%. Hence, to maintain harmonious microclimate, a treatment with humidity was maintained at 65%. Though, it is an innovative approach to study the high temperature effect on reproductive organs under in vivo conditions in tree crops like coconut, it is quite arduous as it entails frequent climbing of trees for adjusting chamber temperature with ambient changes and for pollination of receptive flowers. On the other hand, the alternative in vitro technique standardized could evaluate the response to a wide range of temperatures at 55% and 65% RH. 

High temperature had a profound effect on the emergence of stigmatic region, nectar secretion and stigma appearance. Nectar secretion was observed in WCT two days pre- and post-stigma receptivity at 29 °C like in any other tall coconut variety [67]. Stigma was shiny, sticky and moist with nectar; and the lobes were wide open. High temperature of 33.7 °C (55% RH) induced early nectar secretion and as a consequence during receptivity, the stigmatic surface was closed, dull and dry, making it less attractive to the pollinators; thus, it had a negative impact on fertilization at high temperature [68].The dryness also affects pollen adherence to the stigma and influences the initial stages of pollen–pistil interactions [50,69,70]. In identical studies on sweet cherry [71], pecan (*Carya illinoinensis*) [63] and peach [48], high temperature had significantly reduced stigmatic receptivity. However, it is encouraging to note that, under high humidity, there was less discoloration of stigmatic surface and extended nectar production until the receptivity of stigmas, suggesting alleviation of the high temperature effect to a large extent. 

Further to the effect on stigma receptivity, high temperature also significantly reduced pollen tube expansion through the pistil till it reached ovule. Under in vivo conditions, when morning temperature, coinciding with the time of pollination, rose from 29.5 to 35.5 °C, there was no tube expansion at 55% RH. At 65% RH, there was a small growth of 1.88 mm. An almost similar response pattern was observed under in vitro conditions when the pollinated female flowers were exposed to high temperatures. The reduction in pollen tube expansion was significant at 31 °C with 55% RH compared with that at 35 °C with 65% RH. Pollen germination and pollen tube expansion even under an in vitro nutrient medium were found to be negligible beyond absolute T_max_ > 35 °C in most of the coconut varieties [57], confirming that pollen tube expansion is highly sensitive to high temperature under field conditions. This could plausibly explain the lower fruit set in inflorescences whose opening coincides with those months having a greater number of days with absolute T_max_ > 33 °C in field grown coconut palms [10,11,12]. Likewise, in cherry, moderate increase of the average temperature of 1–3 °C at bloom was sufficient to drastically reduce fruit set [71]. This critical knowledge generated is crucial to understand the impact of environmental factors on pollen germination, pollen tube growth and nut set in coconut. Although not measured in this study, the number of pollen grains produced, pollen dehiscence from anthers and pollen load on the stigma plays an important role in signaling pathways and fertilization. These processes need further investigation to understand their impact on nut set.

These findings alert the crop production specialists about the potential negative effects of even a slight increase in temperature coinciding with flower blooming, which nowadays is on the increase due to climate change and climate variability, especially in coconut-growing regions in the east coast of India [4]. Both under in vivo and in vitro experimental setup, it was observed that under high humidity (65 ± 5%), the effect of high temperature on pollen tube expansion was less, suggesting that in coastal belts where the humidity is high the negative effect of increasing temperature may be minimal, while in interiors where the weather is dry it may have a more severe effect.

## 4. Materials and Methods 

### 4.1. Experimental Location and Plant Material

The progamic phase of West Coast Tall (WCT) coconut and the influence of temperature were evaluated at the research farm of the Indian Council of Agricultural Research—Central Plantation Crops Research Institute (ICAR-CPCRI, Kasaragod, Kerala, India) located at 12°18′ N latitude and 75° E longitude, and at an altitude of 10.7 m above mean sea level. The palms were growing under ambient condition with monthly average T_max_ of 34 °C, T_min_ of 24 °C (Figure 1), relative humidity of around 70% (Figure 1) and mid-day photosynthetically active radiation (PAR) of 1200 µmol m^−2^ s^−1^. WCT, like any other coconut variety, on an average produces one inflorescence each month. The WCT inflorescence is 1–2 m long with 30 or more rachillae bearing 200–300 male flowers, while the bottom few rachillae bear one to two female flowers toward the base (Figure 1).There is an asynchrony in flower opening as the male phase (sequential opening of male flowers) begins first and lasts for about 19 days and the female phase (opening of the female flowers) starts about 3–4 days after completion of the male phase, but lasts for 3–5 days. Female flowers (called ‘buttons’) become receptive between 08:30 and 09:30 depending on the climatic condition and stay receptive for 1–3 days. For this experiment, pollination was done artificially by collecting the pollen from palms of the same variety growing in the same field under identical environments. 

In this experiment, three healthy WCT palms (8–10 years old) with heights ranging from 10 to 12 m were selected. Soon after the opening of the inflorescence, it was emasculated by removing all the male flowers, leaving the spikelets intact (Figure 2a). Emasculated inflorescence was bagged using pollination bags (cloth bags), six days before any of the female flowers became receptive, by an experienced pollinator. As soon as the flower became receptive, the stigma was pollinated by dusting viable pollen, which were collected from either the same palm or adjacent palms, and tested for viability as described earlier [57]. A pollinator climbed the tree daily to pollinate the successive receptive flowers, until all the flowers in the inflorescence were pollinated, keeping a record of each pollination date. As soon as the stigma of all female flowers necrosed or turned brownish-black, approximately on the fifth day after the last pollination, the pollination bag was removed from the inflorescence.

### 4.2. Determination of Progamic Phase 

#### 4.2.1. Dissection and Staining of Pollinated Flowers

The female flowers (*n* = 6; two flowers from each of 3 trees) fertilized under in vivo conditions were collected initially at 2 h and later at 24 h intervals (pollen germination on stigma occurred only after 24 h of pollination) and brought to the laboratory for further observation. Flowers were carefully dissected using a sharp blade and needle to extract the stigma along with the tubular canal and intact ovary (Figure 9). It was then cut longitudinally into thin sections and fixed in a modified Carnoy’s fluid (absolute alcohol:chloroform:glacial acetic acid, 6:4:1) for a period of 12 h.

Histological observations were performed by adopting the quadruple staining method [59]. The staining mixture consists of lactic acid,80 mL; 1% aqueous malachite green, 4 mL; 1% aqueous acid fuchsin, 6 mL; 1% aqueous aniline blue, 4 mL; 1% orange G in 50% alcohol, 2mL; and chloral hydrate, 5 g. Pistils fixed in modified Carnoy’s fluid were hydrated in descending alcohols, transferred to the staining mixture and kept for 24 h at 45 ± 2 °C. Pistils were then placed in a clearing and softening fluid containing 78 mL lactic acid, 10 g phenol and 2 mL 1% orange G, but devoid of chloral hydrate, for 24 h at 45 ± 2 °C, followed by hydrolysis in the clearing and softening fluid at 58 ± 2 °C for 30 min. Following hydrolysis, the specimens were washed twice in lactic acid and immediately mounted on a slide for examination under a stereomicroscope or stored in lactic acid for later assessment.

#### 4.2.2. Microscopic Observations

The specimens were observed under 8X, 3X, 2X and 1.5X magnification objectives of a Nikon SMZ800N stereo microscope (Nikon Corporation, Tokyo, Japan) paired with a Nikon DS-Ril camera. The photographs of the observed microscopic fields were taken with the help of NIS (Nikon Instrument Software) Element D version 4.3. Measurements were performed using ‘Measurement and Annotations’ toolbar of NIS Element. Pollen tube length was measured in micrometers using the polyline length measurement tool of the same software.

### 4.3. High Temperature Treatments of Female Flowers during Receptivity

#### 4.3.1. In Vivo Setup

**Figure 10:**
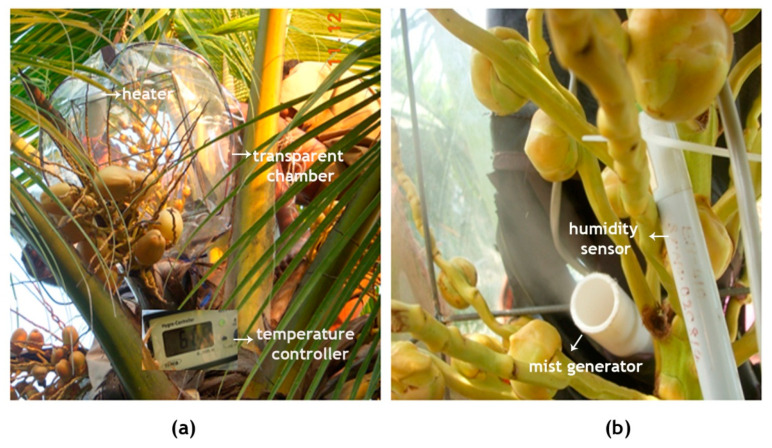
In vivo experimental setup for the induction of high temperature treatment of the inflorescence of the coconut variety WCT. (**a**) The inflorescence was enclosed in a transparent chamber fitted with a microprocessor-controlled heater and (**b**) a mist generator for maintaining the temperature and humidity inside the chamber, respectively.

A temperature simulation chamber, spacious enough to accommodate the coconut inflorescence, was made of transparent plastic of 0.40 mm thickness (Figure 10), enclosed on a skeletal support frame made of mild steel (MS) rods with a diameter of 5 mm and dimensions of 40 cm × 30 cm × 30 cm. The chamber had a front transparent polyvinyl chloride (PVC) flap (that can be opened and closed through a zipper mechanism) and two pockets on its sides for performing the pollination. A few small holes were made on the lower surface of the chamber for smooth exchange of gases and to drain out any moisture inside the bag. A readymade 1000 watts heater (Panasonic, EH-ND12-P, Osaka, Japan) with a speed control regulation was fitted on the frame. The fan fitted in the chamber ensured heat distribution inside the chamber so that every part of the inflorescence underwent uniform heating. Temperature inside the chamber was continuously monitored by a digital PID (Proportional Integral Derivative) controller with a 1-m Pt-100 resistance temperature detector (RTD) sensor.

An ultrasonic mist generator (wave frequency, 70 kHz) was employed to ensure appropriate humidity inside the chamber. The chamber humidity was set at 65 ± 5% hysteresis and a capacitive-type humidity sensor was fitted in the chamber and connected to the humidity controller. When the humidity level declined below the preset value, the ultrasound mist generator switched on and fine mist was sprayed into the chamber. The controller automatically switched off the mist generator when the set RH value was reached.

#### 4.3.2. Treatments

For the in vivo experiment, 12 WCT trees with identical, freshly opened inflorescence were selected. All the male flowers were emasculated. Six days before the female flowers were to become receptive, one inflorescence each from three trees was covered with cloth bags and used as the ambient control (T1). For the rest of the nine trees, the transparent chamber was slipped over the emasculated inflorescence with its opening coming down over the base of spadix (Figure 10) and the opening fastened using a cord. Out of these, three trees with the chamber were used for the chamber control treatment (T2). Similarly, in another set of three trees, temperature inside the chamber was increased by 3 °C (T3) from the chamber control. With this increase in temperature, it was observed that the chamber humidity fell to around 55 ± 5%, from ambient 65 ± 5%. Hence, in the third set of three palms, along with the increase in temperature, humidity was maintained at 65 ± 5% (T4). The treatment imposition was started as soon as the inflorescence was enclosed inside the chamber from 08:00 to 17:00. At bi-hourly intervals, the temperature of the inflorescence with and without the chamber was measured using a Fluke IR thermometer. As and when the flowers under these treatments became receptive, they were pollinated through the side openings of the chamber. The temperature treatment was continued until five days after the last flower was pollinated. In these treatments, observations like the temporal variation in the secretion of nectar, stigmatic appearance and date of female flower receptivity were documented. For histological observations, at least two flowers from three trees of each treatment were collected daily for 4 days after pollination to study pollen germination on stigmatic surface and pollen tube growth through the pistil.

#### 4.3.3. In Vitro Experiment

**Figure 11:**
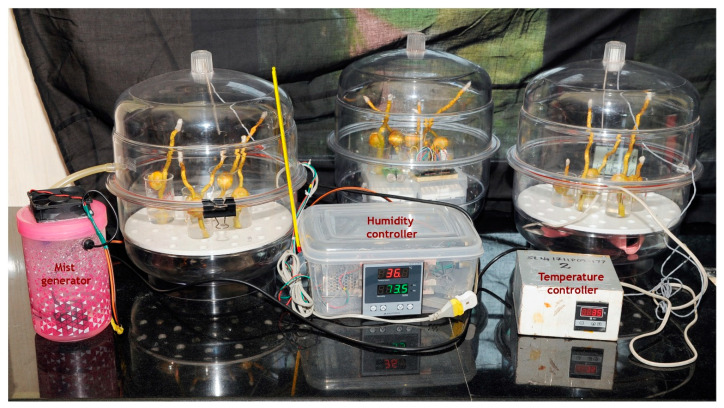
In vitro experimental setup to study the progamic phase of pollinated female flowers of the coconut variety WCT under high temperature and humidity treatments. From left: growth chambers having high temperature and humidity control, control chamber (room temperature and humidity) and high temperature chamber.

Considering the inherent hurdles of the in vivo experimental setup on tree tops and in maintaining multiple temperature profiles, an in vitro setup was developed to test the reproductive response of coconut to a wide range of temperatures (Figure 11). Fresh receptive female flowers were cut from the spadix along with a portion of the rachilla and immediately placed in a beaker containing a media of 10% sucrose, 0.01% boric acid and 1% agar. They were pollinated by WCT pollen and incubated in chambers with set temperatures of 29, 31, 33, 35, 37 and 40 °C with (55% and 65%) humidity control. The chambers used were transparent desiccators with a provision for a small outlet for air flow, fitted from the inside, and an Arduino-based humidity-cum-temperature sensor (DHT 22).The heating source and the mist generator to maintain humidity were as described in the in vivo experiments. The temperature and humidity treatment of the pollinated flowers was started as soon as they were placed in the chamber from 08:00 to 17:00 and continued for a period of 5 days. The incubated flowers were collected daily for four days for histological observations. Each treatment had at least three replications.

### 4.4. Statistical Analysis

In vivo studies involved one inflorescence each from the three trees. For microscopic observations, at least two female flowers from each inflorescence were sampled. Thus, there were six samples for each treatment. For in vitro studies, there were at least three replications for each treatment. All treatment means were analyzed using one-way analysis of variance (ANOVA) in SAS version 9.3 for Windows. Prior to ANOVA analysis, the data were tested for normality and homogeneity of variances based on these two tests, and no transformation was required. Differences between the treatment and control means were determined at the 5% significance level according to Duncan’s multiple range test.

## 5. Conclusions

This study deciphered the progamic phase of the reproductive stage in the coconut variety WCT. In field conditions, the pistil comprising stigma, tubular canal and ovary of a tall variety of coconut is 18.2 ± 4.9 mm in length. The pollen germination on receptive stigma occurs 24 h after pollination and the pollen tube expansion from stigma to ovule requires four days at 29 °C. High temperatures above 33.7 °C advance nectar secretion and stigma receptivity, coupled with dull and dry stigmatic surface during receptivity, making it not only unattractive for pollinators, but also causing shifts in the flowering phenology (early receptivity) that could severely disrupt plant–pollinator interactions in a cross-pollinated crop coconut. The in vivo and in vitro temperature induction systems developed effectively aided in exposing the coconut female flower to the set temperature and revealed that temperatures more than 33.7 °C significantly reduced pollen tube growth and thus negatively impacted nut set. This improved understanding of physiological knowledge of influence of temperature on the progamic phase and protocols (methods) to impose temperature stress on coconut inflorescence is crucial to better comprehend and quantify the impact of changing climate and climate variability on coconut production, and to improve the breeding programs that are focused on screening coconut genotypes and developing high temperature stress-tolerant genotypes.

## Figures and Tables

**Table 1:** Effect of high temperature stress on female flower receptivity and the visual changes observed at the stigmatic surface of the field-grown coconut variety WCT. The inflorescence was exposed to temperature and RH treatments six days before flowers were to become receptive and the visual observations were recorded daily. Data are presented as mean ± SD of female flowers from three inflorescences.

Treatment	Number of Female Flowers per Inflorescence	Number of Receptive Female Flowers	Percent of Non-Receptive Flowers	Changes Observed on the Stigma Surface
Ambient control	19 ± 1.48	18 ± 1.52 ^a^	5	Tiny drops of nectar were observed from the orifice 2–3 days pre- and post-receptivity. Stigma tip, which was shiny and sticky at receptivity, turned dry and dull a few hours post-pollination.
Chamber control	19 ± 2.23	17 ± 1.30 ^a^	10	Similar to ambient condition, except that stigma of a few flowers became dry during receptivity
Chamber with 3 °C rise in temperature and 55 + 5% RH	18 ± 1.20	13 ± 1.92 ^b^	28	High temperature induced early nectar secretion (a few hours after exposure) and during receptivity, stigma was devoid of nectar and the surface was dry.
Chamber with 3 °C rise in temperature and 65 + 5% RH	20 ± 1.14	16 ± 1.14 ^a^	20	A few flowers had nectar during receptivity, and the stigma of a few flowers was shiny, while others were dry.
LSD at 5%	NS	2.01		
*p*-Value	0.141	0.0003		

LSD represents least significant difference between treatment means at 5%. Different letters in each column after the mean values are significantly different at 5% level according to Duncan’s multiple range test. Significance levels of each factor are indicated by *p*-values. NS, non-significant.

**Table 2:** In planta mean pollen tube growth (mm) ± SD (*n* = 6) through pistil of the coconut variety WCT under ambient, chamber control and chamber control with a rise in temperature of 3 °C with 55 ± 5% RH and 65 ± 5% RH. The flowers were artificially pollinated as and when they became receptive and pollinated flower samples were taken daily for documenting pollen tube growth under all the treatments.

Treatment	Days after pollination			
	One	Two	Three	Four
Ambient control	0	3.24 ± 0.43 ^a^	13.96 ± 0.95 ^a^	19.73 ± 0.30 ^a^
Chamber control	0	2.65 ± 0.37 ^a^	9.43 ± 1.63 ^b^	12.89 ± 1.24 ^b^
Chamber with 3 °C rise in temperature at 55 ± 5% RH	0	0.00 ^b^	0.00 ^c^	0.00 ^d^
Chamber with 3 °C rise in temperature at 65 ± 5% RH	0	0.32 ± 0.29 ^b^	0.85 ± 0.07 ^c^	1.88 ± 0.83 ^c^
LSD at 5% *p*-Value	-	0.555 <0.0001	0.040 <0.0001	0.011 <0.0001

LSD represents least significant difference between treatment means at 5%. Different letters in each column after the mean values are significantly different at 5% level according to Duncan’s multiple range test. Significance levels of each factor are indicated by *p*-values.

**Table 3:** Mean pollen tube growth (mm) ± SD (*n* = 3) through the pistil of the coconut variety WCT under in vitro temperature treatments. The fresh receptive flowers collected were artificially pollinated and incubated at different temperatures and two humidity levels. Samples under different treatments were collected daily for histological observations.

Temperature (°C)	55% RH			65% RH		
	Day 2	Day 3	Day 4	Day 2	Day 3	Day 4
29	3.99 ± 0.165 ^a^	13.87 ± 0.813 ^a^	19.01 ± 0.813 ^a^	3.99 ± 0.165 ^a^	13.87 ± 0.813 ^a^	19.07 ± 0.387 ^a^
31	3.39 ± 0.175 ^b^	5.11 ± 0.206 ^b^	8.64 ± 0.866 ^b^	3.67 ± 0.226 ^a^	12.14 ± 0.235 ^b^	19.15 ± 0.284 ^a^
33	3.57 ± 0.207 ^b^	4.93 ± 0.522 ^b^	7.04 ± 1.49 ^c^	3.67 ± 0.226 ^a^	12.14 ± 0.235 ^b^	19.14 ± 0.284 ^a^
35	1.11 ± 0.014 ^c^	1.15 ± 0.045 ^c^	1.22 ± 0.088 ^d^	3.21 ± 0.297 ^b^	6.19 ± 0.229 ^c^	9.88 ± 0.036 ^b^
37	0.91 ± 0.094 ^c^	1.17 ± 0.132 ^c^	1.21 ± 0.167 ^d^	1.22 ± 0.009 ^c^	2.64 ± 0.092 ^d^	3.81 ± 0.029 ^c^
40	0	0	0	0.34 ± 0.050 ^d^	0.64 ± 0.097 ^e^	1.42 ± 0.293 ^d^
LSD at 5% *p*-Value	0.270 <0.001	0.812 <0.001	1.446 <0.001	0.341 <0.001	0.667 <0.001	0.459 <0.001

LSD represents least significant difference between treatment means at 5%. Different letters in each column after mean values are significantly different at 5% level according to Duncan’s multiple range test. Significance levels of each factor are indicated by *p*-values.

## Abbreviations

ET	Elevated temperature
PAR	Photosynthetically active radiation
RH	Relative humidity
T_max_	Maximum temperature
T_min_	Minimum temperature
T_opt_	Optimum temperature
WCT	West Coast Tall
